# Pairwise diversity and tMRCA as potential markers for HIV infection recency

**DOI:** 10.1097/MD.0000000000006041

**Published:** 2017-02-10

**Authors:** Sikhulile Moyo, Eduan Wilkinson, Alain Vandormael, Rui Wang, Jia Weng, Kenanao P. Kotokwe, Simani Gaseitsiwe, Rosemary Musonda, Joseph Makhema, Max Essex, Susan Engelbrecht, Tulio de Oliveira, Vladimir Novitsky

**Affiliations:** a Division of Medical Virology, Stellenbosch University, Tygerberg, South Africa; b Botswana-Harvard AIDS Institute Partnership, Gaborone, Botswana; c Africa Health Research Institute, School of Nursing and Public Health, University of KwaZulu-Natal, Durban, Republic of South Africa; d Harvard T.H. Chan School of Public Health, Boston, MA, USA; e National Health Laboratory Services (NHLS), Tygerberg Coastal, South Africa; f Research Department of Infection, University College London, London, United Kingdom; g College of Health Sciences, University of KwaZulu-Natal, Durban, Republic of South Africa; h Division of Sleep Medicine, Brigham and Women's Hospital, Boston, Massachusetts.

**Keywords:** HIV incidence, HIV recency, HIV-1C, pairwise distances, tMRCA

## Abstract

Intrahost human immunodeficiency virus (HIV)-1 diversity increases linearly over time. We assessed the extent to which mean pairwise distances and the time to the most recent common ancestor (tMRCA) inferred from intrahost HIV-1C *env* sequences were associated with the estimated time of HIV infection. Data from a primary HIV-1C infection study in Botswana were used for this analysis (N = 42). A total of 2540 HIV-1C *env* gp120 variable loop region 1 to conserved region 5 (V1C5) of the HIV-1 envelope gp120 viral sequences were generated by single genome amplification and sequencing, with an average of 61 viral sequences per participant and 11 sequences per time point per participant. Raw pairwise distances were calculated for each time point and participant using the ape package in R software. The tMRCA was estimated using phylogenetic inference implemented in Bayesian Evolutionary Analysis by Sampling Trees v1.8.2. Pairwise distances and tMRCA were significantly associated with the estimated time since HIV infection (both *P* < 0.001). Taking into account multiplicity of HIV infection strengthened these associations. HIV-1C *env-*based pairwise distances and tMRCA can be used as potential markers for HIV recency. However, the tMRCA estimates demonstrated no advantage over the pairwise distances estimates.

## 1 Introduction

Human immunodeficiency virus (HIV) incidence is critical for monitoring the state of the epidemic, as well as for the design and evaluation of prevention interventions. Recency of HIV infection (time since virus transmission) can be estimated by repeat testing a cohort of HIV-negative participants, although this approach is associated with substantial cost and logistical challenges. The cross-sectional methods based on serological markers have been proposed as a reasonable alternative.^[1–14]^ However, the variability of immune response, diversity of HIV-1 subtypes, and unknown use of antiviral therapy present challenges for serological testing.

The diversity of viral quasispecies can be assessed through the distribution of pairwise distances, or by computing the frequency of ambiguous (mixed) calls. During the transition period from early to chronic HIV-1 infection, the diversity of viral quasispecies within the host increases almost linearly.^[15–17]^ This observation provides an opportunity to apply pairwise distances inferred from the intrahost virus sequences to estimate the time since HIV infection (HIV recency).^[1,18–20]^ Approximately 80% of HIV-1 infections are seeded by a single founder strain as a result of the severe bottleneck upon virus transmission.^[21–23]^ Virus sequences representing HIV-1 quasispecies can then be used to reconstruct viral phylogenies and to infer the time to the most recent common ancestor (tMRCA), which may also be useful for estimating HIV recency.^[24,25]^

Recent developments in sequencing technology have enabled the high throughput of intrahost sequences representing virus quasispecies. These developments have also facilitated the application of coalescent analysis and the estimation of the tMRCA for HIV infection recency.^[26,27]^ For example, Giorgi et al^[24]^ have developed a Poisson–Fitter tool that uses a set of homogeneous sequences and performs statistical tests on the Hamming Distance frequency distributions. The tool computes the best fitting Poisson distribution through Maximum Likelihood, performs a Goodness of Fit test, and tests for Star-Phylogeny (a Star-Phylogeny assumes that all the species radiated simultaneously from 1 ancestor). However, this tool requires samples from the very early stage of HIV infection (2–5 weeks), under the assumption of homogeneous viral sequences prior to selection pressure and fast exponential growth. Poon et al^[25]^ demonstrated that tMRCA reconstructed by coalescent analysis of longitudinal virus sequences (generated by next-generation sequencing) can be used for the accurate estimation of HIV recency. Park et al^[19,20]^ have suggested an algorithm for identifying signatures of incident, chronic, and multiple infections to overcome a limitation on pairwise distance-based assays, which may potentially misclassify early infections with multiple distinct founder strains as chronic infections.

However, it remains unclear whether HIV-1 pairwise distances and/or tMRCA can be used as either independent or complementary markers of HIV recency in cross-sectional sample, particularly in the context of heterogeneity of individual immune responses, levels of virus replication, variation of HIV-1 subtypes, nonuniform diversity across HIV-1 genes, and different modes of virus transmission. In this study, we addressed whether estimated time of HIV infection could be determined from cross-sectional sampling by utilizing a cohort with known time of seroconversion and prospective sampling. Normally, only a single-per-person sample is available in many population-based surveys. To assess the utility of pairwise distances and tMRCA in estimating the time since infection in cross-sectional sampling within the early stage of a predominantly heterosexual HIV-1C epidemic in Botswana, we took advantage of the availability of a unique set of samples with known time since infection. We used longitudinal samples as a source of virus sequences representing HIV quasispecies over time as a reference set, and used appropriate statistical techniques to account for multiple sampling from the same individuals by applying a mixed-effects model.

## 2 Materials and methods

### 2.1 Study participants

A total of 42 participants recruited into a primary HIV infection cohort in Botswana (Tshedimoso Study) with longitudinal sampling and estimated time of seroconversion were included.^[28]^ The time of HIV infection was considered to be about 14 days prior to seroconversion.^[29,30]^ HIV-1C *env* gp120 variable loop region 1 to conserved region 5 (V1C5) of the HIV-1 envelope gp120 viral sequences were generated by single genome amplification and sequencing, as described elsewhere.^[31,32]^ A total of 2540 sequences represented an average of 61 viral sequences per participant and 11 sequences per time point per participant. The analyzed region of *env* V1C5 corresponds to nucleotide positions 6615 to 7757 relative to the HXB2 reference strain. The study design and participant characteristics were described elsewhere.^[31,33,34]^ Briefly, study participants were predominantly female (76.2%), with a median age of 27 (interquartile range 25–32.5) years at enrollment.

All participants were infected with HIV-1C. Both viral RNA and proviral DNA were used as templates for amplification and sequencing. The accession numbers of the viral sequences used in this study are KC628761–KC630726. The viral mutations within the targeted V1C5 region of HIV-1C *env* gp120 using serial samples have been described elsewhere.^[35,36]^

The initial set included 223 time points. In the preliminary analysis, we used phylogenetic inference to identify time points with evidence of HIV-1C super-infection based on branching patterns and presence of phylogenetically distinct clusters separated by other participants’ sequences. Based on this analysis, 4 time points from 2 participants with evidence of super-infection were excluded from analysis. In addition, 15 time points were excluded due to the initiation of ART. Because virologically suppressed individuals are unlikely to represent recent HIV infection, and are associated with false-recent infections,^[9,37]^ 35 time points with HIV-1 RNA load below 1000 copies/mL were also excluded from analysis. The final reference set of viral sequences comprised 164 time points from 42 participants spanning over 2 years after estimated time of HIV infection/seroconversion. For a subanalysis, we excluded an additional 19 time points with evidence of subclusters (see section “Test for clustering” below). These cases were assumed to represent transmissions of multiple viral variants from the same (or closely related) source(s) of established (chronic) HIV infection. The sample set in the subanalysis therefore included 145 time points from 42 participants.

This study was conducted according to the principles expressed in the Declaration of Helsinki. The study was approved by the Health Research and Development Committee (HRDC) in Botswana (Protocol number PPME-13/18/1) and the Office of Human Research Administration (OHRA) of the Harvard School of Public Health (Protocol number 10491). All participants provided written informed consent.

### 2.2 Pairwise distances

Uncorrected (raw) pairwise distances were calculated using the ape package in R.^[38]^

### 2.3 Recombination analysis and phylogenetic inference

Each set of viral sequences per time point per participant was checked for potential recombination by the RDP v.4 package.^[39]^ Sequences with evidence of recombination signal based on at least 2 out of 7 methods in the RDP package were excluded before the test for clustering was applied. Phylogenetic tree reconstruction was performed using a maximum likelihood (ML) framework, with PhyML^[40]^ as implemented in SeaView v2.4 software^[41]^ using the GTR + G + I model.

### 2.4 Test for clustering

For each pool of viral sequences per participant per time point, the pairwise distance matrix was generated by dist.dna (ape package in R) using multiple sequence nucleotide alignment. To identify potential clusters within the pool of viral sequences, kmeans (stat package in R) was utilized and partitioning of the pairwise distance matrix into 2 groups (k = 2) was performed. The ratio of withinss (vector of within-cluster sum of squares, 1 per cluster) to betweenss (the between-cluster sum of squares) was used to determine the validity of partitioning. The clustering was considered valid if the ratio values (withinss to betweenss) for both tested clusters were greater than zero and less than 0.2. This range corresponds to the monophyletic lineage of viral sequences with subclustering, which is evident from a combination of the relatively long branches separating clusters of viral sequences and short branches within each cluster. This branching topology was assumed to be associated with transmission of multiple viral variants from the same (or closely related) source(s) of established (chronic) HIV infection.

### 2.5 Estimating tMRCA

The tMRCA was estimated for each time point per participant using Bayesian Markov Chain Monte Carlo (MCMC) phylogenetic inference implemented in Bayesian Evolutionary Analysis by Sampling Trees v.1.8.2 package.^[42]^ Each independent run had a chain length of 100,000,000 with a sample frequency of 10,000. The previously estimated intrahost rate of nucleotide substitution per site within the HIV-1 gp120 V1C5 region (1.58 × 10^−2^) was used.^[32]^ To avoid over-parameterization, all runs were performed using the HKY substitution model with a gamma distributed rate variation, a strict molecular clock model, and a constant population size tree prior. The MCMC log output of each run was examined in Tracer v1.6^[43]^ to verify convergence and effective sample sizes (ESS) greater than 200. Mean tree model root height was used to determine the time since infection along with the 95% highest posterior density (HPD) intervals.

### 2.6 Statistical analysis

Our study benefits from a high frequency of HIV testing and longitudinal sampling^[28]^ which enabled us to estimate the time since infection, measured in days since seroconversion date plus 14 days.^[29]^ Because the time of infection was estimated with high precision, we could then compare the accuracy of the pairwise distances and tMRCA.

We used a linear mixed-effects model to assess the association between estimated time since infection and tMRCA or raw pairwise distances, taking into account the intrahost dependency of repeated measurements. We calculated the accuracy (sensitivity and specificity) in predicting time from infection using either tMRCA or raw pairwise distances in categorizing participants within X (X = 130, 160, 360) days from infection. The sensitivity and specificity for tMRCA are defined as P(predicted time from infection using tMRCA < X days | estimated time from infection < X days) and P(predicted time from infection using tMRCA ≥ X days | estimated time from infection ≥ X days). These quantities for raw pairwise distance are defined in the same way. Confidence intervals were obtained using the bootstrap method, treating each participant as a sampling unit and basing on 100 bootstrap samples. Analyses were performed using data within 2 years of infection and based on measurements from 164 time points from 42 participants. Analyses were repeated in a subset of measurements excluding 19 time points with evidence for subclusters (see section “Test for clustering” above). Mixed-effects models were fit using R version 3.2.3. All other analyses were performed using SAS 9.4 (Cary, NC). All *P*-values were 2-sided. *P*-values < 0.05 were considered statistically significant.

## 3 Results

**Figure 1:**
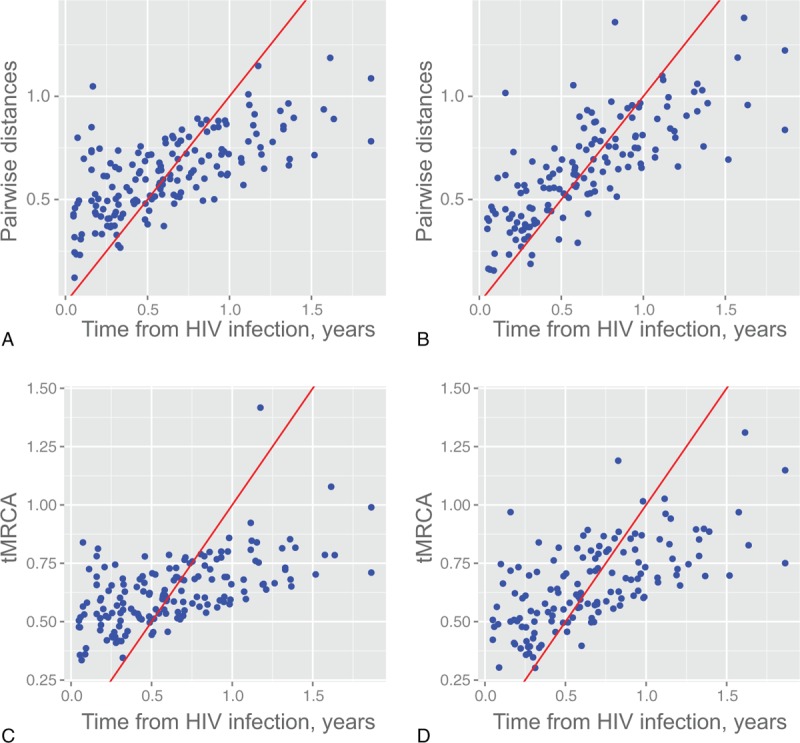
Predicted times from infection using pairwise distance (top panels) or the time to the most recent common ancestor (tMRCA, bottom panels) versus estimated times from infection. The panels on the left represent predictions using all 164 measurements and those on the right represent predictions using a subset of 145 measurements excluding those with evidence for subclustering. The red lines are 45° lines passing origin, reflecting perfect prediction.

We investigated whether pairwise distances of viral intrahost sequences and reconstructed tMRCA could be used as markers of time since infection in a predominantly heterosexual HIV-1C epidemic. The estimated time since infection was significantly associated with tMRCA (**β **= 0.237; *P* < 0.001) and pairwise distances (**β** = 27.6; *P* < 0.001; Table 1). Excluding subclustering resulted in a stronger association with the estimated time since infection for both tMRCA (**β **= 0.435; *P* < 0.001) and pairwise distances (**β **= 43.8; *P* < 0.001; Table 1). Pairwise distances and tMRCA tend to overestimate time since infection at the early time points and overestimate at the later time points (Fig. 1A and B). Excluding time points with evidence of subclusters seemed to mitigate this trend (Fig. 1C and D).

**Table 1:**
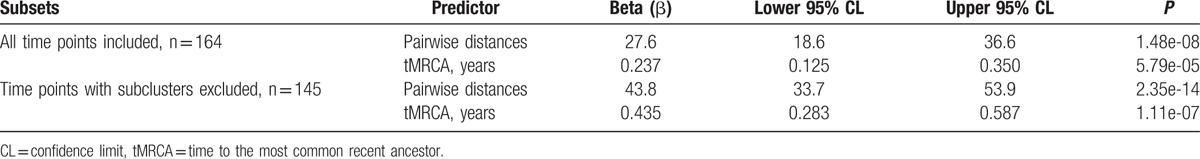
Associations between analyzed parameters using linear mixed-effect model.

We calculated the sensitivity and specificity of using pairwise distances or tMCRA to predict time since infection within 130, 180, and 360 days (Table 2). Consistent with the trends seen in Fig. 1 that predicted times using either tMRCA or raw pairwise distance tend to overestimate times from infection in earlier time points but underestimate those in later time points, the sensitivity was lower for earlier time points and higher for later time points, whereas the specificity was higher for earlier points and lower for earlier time points. Both tMRCA and raw pairwise distances had high sensitivity (but lower specificity) at the 360-day threshold.

**Table 2:**
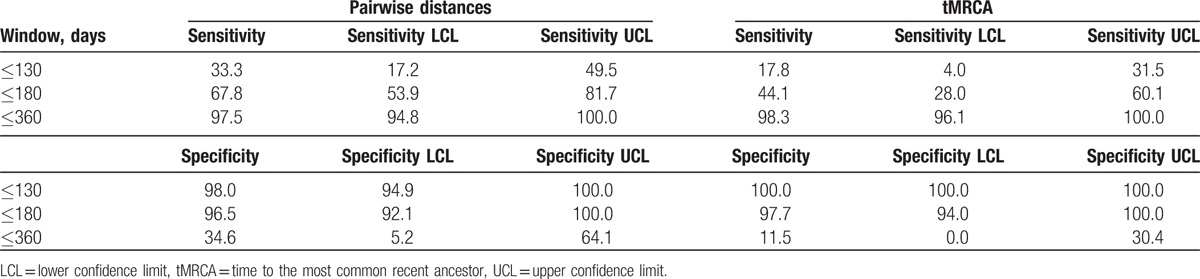
Sensitivity and specificity of pairwise diversity and tMRCA estimate for thresholds 130, 180, and 360 days since infection.

## 4 Discussion

Estimation of HIV incidence is crucial for understanding the trends and dynamics of the HIV epidemic, monitoring of HIV incidence, and designing and evaluating preventions and interventions.^[44–48]^ In this study, we assessed whether pairwise diversity of intrahost HIV sequences and inferred tMRCA could be used as potential markers for the estimation of HIV recency. We found that both pairwise distances and tMRCA inferred from intrahost HIV-1C *env* gp120 V1-C5 sequences (representing single time points) correlated directly with the estimated time of HIV infection. We used only a single time point for each pool of viral sequences, as this is the typical case for the vast majority of epidemiological studies interested in estimating HIV recency from cross-sectional sampling.

The diversity of the HIV-1 *env* gene increases linearly at approximately 1% per year during the early stage of HIV-1 subtype B infection,^[16]^ particularly when the transmitted/founder virus is represented by a single viral variant. Therefore, pairwise distances can be used as a marker of HIV recency for infections with a single transmitted virus. However, HIV infections with multiple transmitted viruses, even in the early stage of HIV infection, could have elevated levels of viral diversity which are associated with established/chronic HIV infection.^[19]^ Thus, recent HIV infections with multiple transmitted viruses could be misclassified as chronic HIV infections. In this study, we demonstrated that controlling for multiplicity of HIV infection improves association between pairwise sequence diversity and the estimated time of HIV infection.

We found no advantage of tMRCA as a marker of HIV recency (in comparison with pairwise distances). It is possible that advanced evolutionary models applied selectively to each pool of viral sequences could improve accuracy in the estimation of tMRCA. Both markers, tMRCA and pairwise distances, are significantly associated with time from HIV infection, suggesting the potential utility of intrahost virus sequences for HIV recency estimation in cross-sectional sampling. However, using either marker alone may not be adequate to predict time from infection. Further research investigating combining information from multiple sources may help improve the prediction accuracy.

The study has limitations. We used the available set of intrahost HIV-1C *env* sequences, which does not necessarily adequately represent the distribution of HIV-1C viruses on a population level. All specimens were collected in 2004 to 2010, and therefore might not reflect the current HIV epidemic in Southern Africa, as circulating viruses could differ over time. The sample set was enriched with early time points, with a relatively small number of later time points. To avoid over-parameterization, we used a relatively simple evolutionary model and simplistic parameters for the estimation of tMRCA, which is another study limitation. To optimize parameters in the Bayesian Evolutionary Analysis by Sampling Trees analysis for a more accurate estimation of tMRCA, further studies are warranted. Our preliminary data suggest that each pool of viral quasispecies might require individual optimization of parameters and model selection. In a limited set of preliminary runs, we found that applying more complex models to a pool of viral quasispecies with low level of viral diversity results in poor convergence and unstable behavior of the MCMC run, which is likely to represent a negative effect of over-parameterization.^[49–51]^ It is also possible that using virus sequences representing multiple time points of sampling (if available) could improve the estimation of tMRCA, as described by Poon et al.^[25]^
